# A low meat diet increases the risk of open-angle glaucoma in women—The results of population-based, cross-sectional study in Japan

**DOI:** 10.1371/journal.pone.0204955

**Published:** 2018-10-02

**Authors:** Reiko Kinouchi, Satoshi Ishiko, Kazuomi Hanada, Hiroki Hayashi, Daiki Mikami, Tomofumi Tani, Tatsuya Zenimaru, Motofumi Kawai, Seigo Nakabayashi, Motoshi Kinouchi, Akitoshi Yoshida

**Affiliations:** 1 Medicine and Engineering Combined Research Institute, Asahikawa Medical University, Asahikawa, Hokkaido, Japan; 2 Department of Ophthalmology, Asahikawa Medical University, Asahikawa, Hokkaido, Japan; 3 Department of Ophthalmology, Rumoi Municipal Hospital, Rumoi, Hokkaido, Japan; 4 Zenimaru Eye Clinic, Rumoi, Hokkaido, Japan; 5 Department of Dermatology, Red Cross Asahikawa Hospital, Asahikawa, Hokkaido, Japan; Bascom Palmer Eye Institute, UNITED STATES

## Abstract

**Background:**

Studies identifying modifiable lifestyle risk factors related to open-angle glaucoma (OAG) are limited, especially from Asian countries. This study aimed to identify lifestyle risk factors for OAG in a Japanese population.

**Methods and findings:**

This population-based, cross-sectional study recruited Japanese participants aged 40 years or older from January 2013 to March 2015. We took fundus photographs for OAG screening, determined lifestyle and health characteristics through a questionnaire and performed physical examinations. The participants who had suspect findings in the fundus photographs were sent for a detailed ophthalmic examination to diagnose OAG. Lifestyle and heath characteristics were statistically compared between the OAG and non-OAG participants. A total of 1583 participants were included in the study, of which 42 had OAG and 1541 did not have OAG. The number of days per week that the female participants consumed meat (mean±SD; OAG: 1.7±1.2 days, non-OAG: 2.7±1.5 days) was negatively associated with OAG (OR = 0.61; 95% CI: 0.43–0.88; *p =* 0.007). Higher intraocular pressure was positively associated with OAG in men (OR = 1.20; 95% CI: 1.05–1.38, *p* = 0.009). No significant difference between participants with and without OAG was observed for a range of other lifestyle factors and health criteria including self-report of diabetes, number of family living together, body mass index, blood pressure, pulse rate, coffee drinking, tea drinking, alcohol drinking, number of fruits consumed per day and days of fish consumption per week.

**Conclusions:**

A higher weekly consumption of meat appears to be negatively associated with OAG in Japanese women. Increasing the dietary intake of meat can contribute to reducing the risk of developing OAG.

## Introduction

Globally, 2.1 million people are visually impaired as a result of glaucoma and along with macular degeneration, it is a leading cause of irreversible blindness worldwide [1]. A systematic review found that the global prevalence of glaucoma in individuals aged 40 to 80 years was 3.54% (open-angle glaucoma [OAG]: 2.34%; angle-closure glaucoma: 0.73%) and in 2013, the number of people with glaucoma was estimated to be 64.3 million, with a projected increase to 111.8 million by 2040 [2, 3].

Maintaining a healthy lifestyle and avoiding modifiable disease risk factors is important for reducing the risk of developing many chronic diseases, including OAG. It has been reported that dementia has an association with glaucoma [4, 5] and studies have also shown that about one-third of dementia is attributable to a combination of modifiable risk factors [6]. While high intraocular pressure (IOP) is medically treatable, other established risk factors for OAG, such as a positive family history and high myopia, are scarcely modifiable [2, 7–10]. There are some reports describing modifiable lifestyle risk factors for glaucoma. Heavy smoking, unmarried marital status and low consumption of certain fruits, vegetables and fatty fish were reported as risk factors for OAG [11–14]. However, most of these reports are from western countries and their findings are considered controversial. In order to extrapolate these findings into an Asian cohort, we conducted a population-based study in northern Japan to search for modifiable lifestyle risk factors of OAG.

## Methods

### Study design and participants

**Fig 1:**
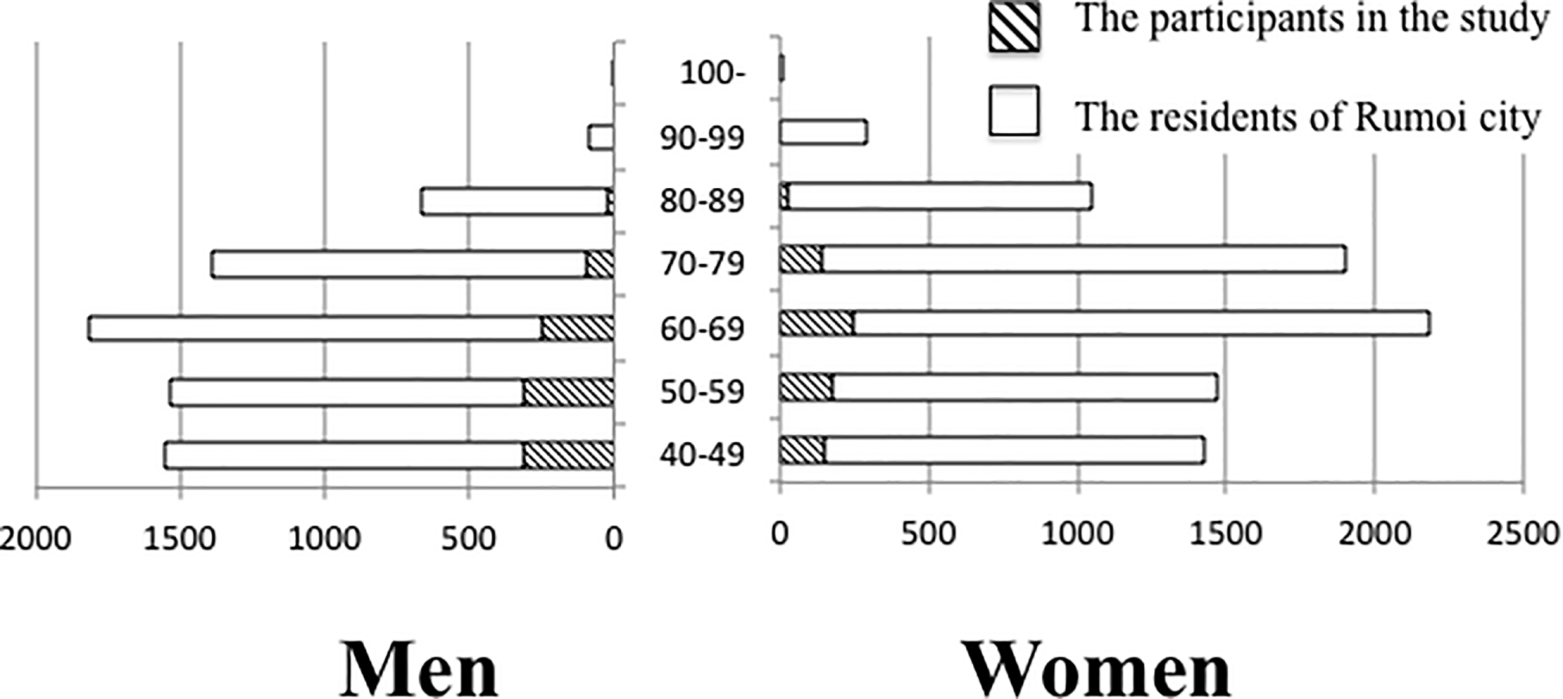
Age distribution of the entire population of Rumoi city and the participants of this study. Age distribution of entire population of Rumoi is based on the information contained in the basic resident register on January 2015. Shaded areas represent the participants involved in the current study. Exact numbers are indicated in S1 Table.

We conducted a population-based cross-sectional study with a screening of fundus photographs from residents aged 40 years or older from Rumoi city. Rumoi is a seaside rural city in Hokkaido prefecture located in northern Japan. We selected Rumoi for this study as the local government has actively cooperated in population-based research studies and there are limited ophthalmic clinics in the city. We took fundus photography and physical examinations at the Rumoi health station which was established by Rumoi city to improve the citizen’s health. The study was carried out in accordance with the tenets of the Declaration of Helsinki and it was approved by the research ethics committee of Asahikawa Medical University. Participants were recruited using the city's public relations magazine, posters and direct phone calls to businesses and public offices located in the city. 1731 participants from 15373 residents (based on the Rumoi city resident register as of January 2015) were recruited for this study from January 2013 to March 2015 (Fig 1; S1 Table). This equated to 11 percent of the residents aged 40 years or older.

### Procedures

After obtaining written informed consent, we checked the participants’ prescription record and asked them to complete a questionnaire detailing their occupation, lifestyle habits and health information, including any present illness (S1 Text). They also undertook a physical examination which included measurement of height, body weight and systolic and diastolic blood pressure. In addition, pulse rate was measured using the UDEX-APG1 automatic electronic sphygmomanometer with pulse wave meter (Wellup, Kanagawa, Japan), body composition was assessed using the MC-190 multi-frequency segmental body composition analyzer (Tanita, Tokyo, Japan) and IOP was measured using the NT-5P non-contact tonometer (Nidek, Aichi, Japan). Fundus photographs were taken using an AFC-230 non-mydriatic auto fundus camera, which has a resolution of 21.1 million pixels (Nidek, Aichi, Japan). All these findings were uploaded to the Wellnet Link health information system, which is managed by Asahikawa Medical University Telemedicine Center. Fundus photographs were reviewed by ophthalmology specialists (authors RK, SI, KH) and the participants were then classified into three groups as follows: no abnormality detected or some changes found but “no detailed examination needed”; some abnormality suspected and “detailed examination required”; “undeterminable”, which meant the fundus photographs were unclear to evaluate. Participants were informed of their results through Wellnet Link or told directly at the Rumoi health station. Results were also mailed to participants who needed a detailed examination. Detailed examinations were conducted at the local hospital or clinic and the results were included in this study. There were two ophthalmologists (authors TT, TZ) working at the local hospital and clinic in Rumoi city. In the detailed examination, visual acuity, slit-lamp biomicroscopic examination, tonometry and fundus examination were conducted. When OAG was suspected based on optic disc, fundus or optical coherence tomography findings, a visual field test with the Humphrey Field Analyzer (Carl Zeiss, Oberkochen, Germany) or Octopus 1-2-3 (Haag-Streit Diagnostics, Koniz, Switzerland) was performed, along with evaluation of the anterior chamber angle. We diagnosed OAG based on both optic disc and visual field findings, independent of IOP [15, 16]. Diagnosis of OAG corresponded to the category 1 diagnostic criteria of the International Society of Geographical and Epidemiological Ophthalmology [15].

### Statistical analysis

**Fig 2:**
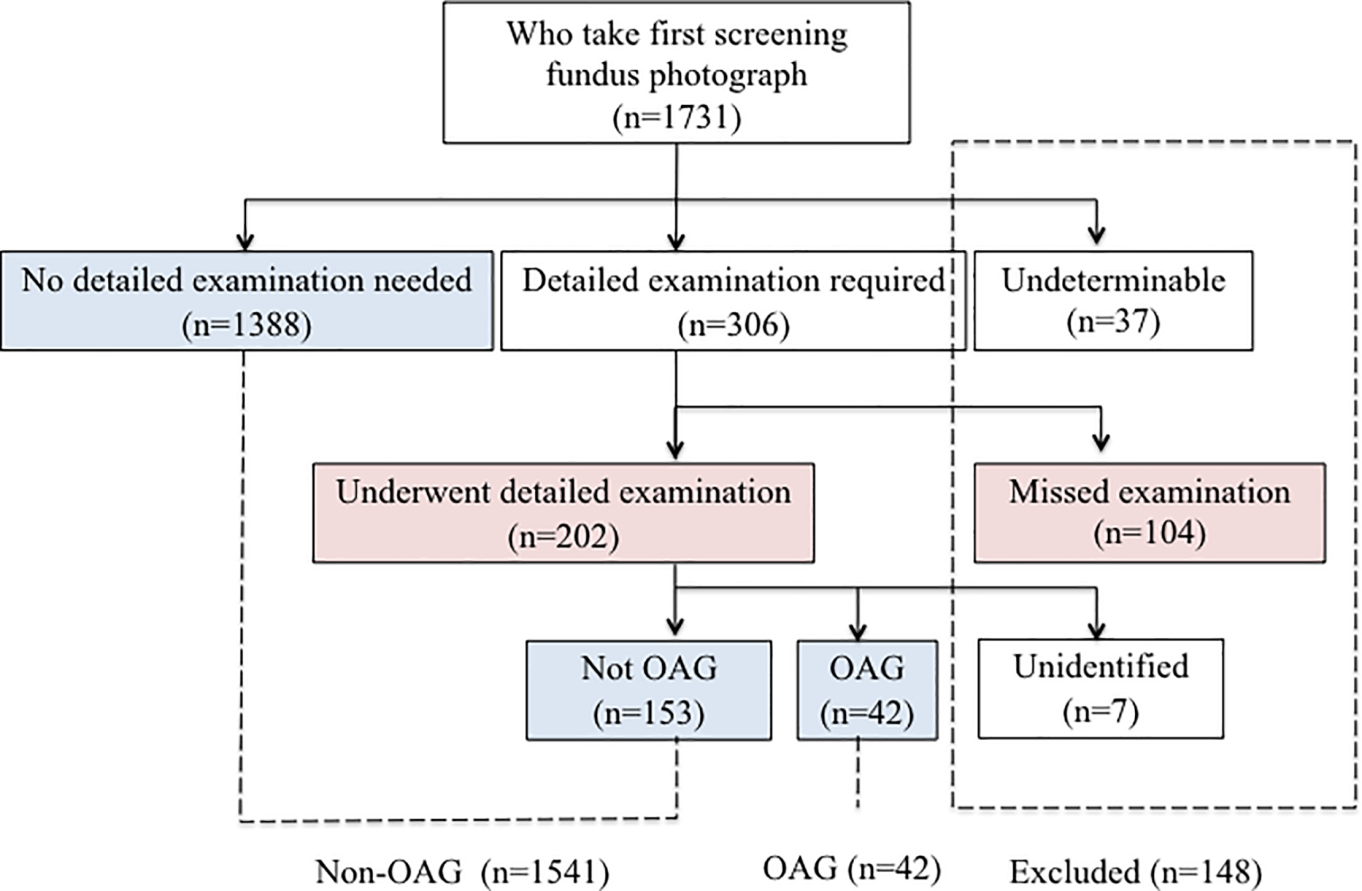
Summary of the participant groups included in the study. Among the 1731 participants, we included 1541 in the control group (non-OAG) and 42 in the OAG group. The participants included in the OAG and non-OAG groups are represented in the blue boxes. We excluded 148 participants who either had unclear fundus photographs, did not undergo a required detailed examination or could not be definitively diagnosed as having OAG. Participants who underwent a detailed examination and those that did not are represented in the pink boxes. We also conducted statistical analysis to compare these two groups.

Statistical comparison of the OAG and non-OAG groups was conducted (Fig 2). For the statistical analyses, R version 3.3.0 (The R Foundation for Statistical Computing Platform, Vienna, Austria) and EZR (Jichi Medical University, Saitama, Japan), a graphical user interface for R, were used. Fisher’s exact test and Wilcoxon rank sum test were utilized for univariate analysis and logistic regression was performed for multivariate analysis. For logistic regression, we entered as variables the previously reported risk factors for OAG (age and hypertension), the factors that had significant association with OAG in the univariate analysis in the current study (meat diet, intraocular pressure and smoking) and the factors associated with whether the participant was eligible for a detailed examination in the current study (occupation status and exercise hours). As the risk of developing OAG could be influenced by gender, we compared the OAG and non-OAG groups in both males and females independently.

To assess whether the study participants who did not undergo a detailed examination could influence the final results, we compared 202 individuals who underwent a detailed examination to 104 individuals who did not (Fig 2). There were significant differences in gender, occupation status and exercise frequency between the two groups (S3–S5 Tables). Therefore, we included occupation status and the amount of daily exercise as variables to adjust the logistic regression comparing OAG and non-OAG participants.

## Results

The age range of the 1731 participants was 40 to 89 years and the age distribution is shown in Fig 1 and S1 Table. We excluded 37 participants who were diagnosed as undeterminable from the fundus photographs, 104 who did not undertake a detailed examination and seven participants whose diagnosis was not conclusive after a detailed examination. A total of 1583 participants (880 men, 703 women) were deemed eligible for the final study cohort, of which 42 (19 men, 23 women) were diagnosed with OAG and 1541 (861 men, 680 women) did not have OAG (Fig 2). The mean age of the men and women was 55.9 ± 10.6 years and 60.1 ± 10.9 years respectively (mean ± SD). Eighty-three percent of the male participants and 49% of the female participants were employed.

Among the 306 participants (186 men, 120 women) who were evaluated as “detailed examination required” following screening of the fundus photographs, 54.8% of the men and 83.3% of the women underwent the detailed examination. The results of the comparative analysis between the participants who underwent a detailed examination and those that did not is shown in S2–S4 Tables. More men than women did not undergo a detailed examination (95% CI: 1.59–5.32; *p* < 0.001) and participants who were employed were also more unlikely to undergo a detailed examination (95% CI: 1.15–4.77; *p* = 0.02; S5 Table). The number of hours spent exercising was positively associated with undertaking a detailed examination by univariate analysis but no significant association was observed by multivariate analysis.

The results of Fisher’s exact test comparing the OAG and non-OAG groups is shown in Table 1. Self-reported hypertension was positively associated with OAG in the cohort with men and women combined (*p* = 0.02). No statistical difference was observed between the OAG and non-OAG groups with self-reporting of diabetes mellitus.

**Table 1:** Results of Fisher’s exact test comparing the OAG and non-OAG groups.

Parameter	OAG		Non-OAG		*P Value*
	n	(%)	n	(%)	
Total men and women	42	-100	1541	-100	
Self-report of diabetes	1	-2	101	-7	0.52
Self-report of hypertension	15	-36	302	-20	0.02
Men	19	-100	861	-100	
Self-report of diabetes	1	-5	64	-7	1
Self-report of hypertension	7	-37	160	-19	0.07
Women	23	-100	680	-100	
Self-report of diabetes	0	0	37	-5	0.73
Self-report of hypertension	8	-35	142	-21	0.12

OAG = open-angle glaucoma

The results of the Wilcoxon rank sum test and the univariate analysis for continuous variables is demonstrated in Table 2. High IOP in each eye was positively associated with OAG in men (*p* = 0.02 in the right eye and *p* = 0.01 in the left eye). The number of days in which meat was consumed per week was negatively associated with OAG in women (*p* = 0.003; OAG: 1.7 ± 1.2 days; non-OAG: 2.7 ± 1.5 days). Smoking (packs/year) was negatively associated with OAG in women (*p* = 0.04). No significant difference between the OAG and non-OAG groups was observed for the following factors: age; number of family members living together; walking hours per day; body mass index; blood pressure; coffee consumption; tea consumption; alcohol consumption; daily intake of fruit; weekly intake of fish. No association with OAG was also found with smoking history (present, past, none) heavy smoking (≥ 40 packs/year), or estimated ocular perfusion pressure (S5 and S6 Tables).

**Table 2:** Results of the Wilcoxon rank sum test comparing the OAG and non-OAG groups.

Parameter			Men								Women						
			OAG			Non-OAG			*P V*alue		OAG			Non-OAG			*P V*alue
			(n = 19)			(n = 861)					(n = 23)			(n = 680)			
Age (yrs)			59.7	±	10.3	55.8	±	10.6	0.11		63.8	±	10.9	60	±	10.9	0.08
Questionnaire																	
	Number of family living together (include oneself)		2.7	±	0.8	2.6	±	1.2	0.43		2.6	±	0.9	2.4	±	1.1	0.16
	Activity																
		Walking (hours/day)	2.6	±	2.4	2	±	2.2	0.11		2.3	±	1.8	2.9	±	2.6	0.47
	Habits																
		Smoking (Pack-year)	19	±	17	23	±	21	0.56		1	±	4	5	±	11	0.04
		Coffee (cups/day)	1.6	±	1.3	2.1	±	1.7	0.32		1.6	±	1.1	1.8	±	1.5	0.77
		Tea (cups/day)	1.2	±	1.6	1.2	±	1.7	0.62		2.6	±	2.2	1.8	±	2.2	0.09
		Alcohol (glasses/day)	0.7	±	1	1.3	±	1.6	0.16		0.3	±	0.5	0.4	±	0.9	0.73
		Fruit (number/day)	0.6	±	0.5	0.6	±	0.7	0.8		0.8	±	0.7	0.9	±	0.7	0.43
		Meat (eating days/week)	2.8	±	1.5	2.5	±	1.4	0.26		1.7	±	1.2	2.7	±	1.5	0.003
		Fish (eating days/week)	2.7	±	1.2	2.9	±	1.6	0.71		4.1	±	1.8	3.5	±	1.7	0.12
Measurements																	
	Body mass index (kg/m^2^)		25	±	2.8	24.7	±	3.6	0.51		23.6	±	4.1	23.3	±	3.9	0.56
	Systolic blood pressure(mmHg/Ag)		137	±	2.8	134	±	19	0.45		128	±	19	127	±	19	0.84
	Diastolic pressure(mmHg/Ag)		81.8	±	2.8	79.6	±	11.8	0.26		74.7	±	10.6	76.6	±	11	0.39
	Pulse rate/minute		74.4	±	2.8	77.2	±	12.7	0.31		77	±	10.3	76.8	±	11.7	0.83
	Estimated values																
		basal metabolism (kcal/day)	1533	±	212	1503	±	193	0.81		1104	±	121	1095	±	120	0.39
		Muscle (kg)	52.9	±	6.3	51.9	±	5.8	0.78		35.9	±	3.1	35.5	±	3.1	0.39
		Bone mass (kg)	2.9	±	0.3	2.8	±	0.3	0.69		2.1	±	0.3	2.1	±	0.3	0.44
		Body fat (%)	15.9	±	5.2	15	±	7.7	0.34		17.7	±	7.7	17.6	±	7.5	0.84
		Body water (%)	38.4	±	5.1	38	±	5	0.97		27.9	±	3.4	27.7	±	3.1	0.32
	Intraocular pressure (mmHg/Ag)																
		left eye	15.6	±	3.1	13.9	±	2.8	0.01	*	14	±	2.9	14	±	2.8	0.9
		right eye	15.6	±	2.7	14.1	±	2.7	0.02	*	14	±	2.9	14.2	±	2.8	0.67
			(mean	±	SD)	(mean	±	SD)			(mean	±	SD)	(mean	±	SD)	

OAG = open-angle glaucoma; SD = standard deviation

The results of the multivariate logistic regression analysis are shown in Table 3. Higher IOP in men (OR = 1.20; 95% CI: 1.05–1.38; *p* = 0.009) and a lower consumption of meat in women (OR = 0.61; 95% CI: 0.88–0.43; *p* = 0.007) were significantly associated with OAG. Smoking (packs/year) was not significantly associated with OAG when adjusted for other variables in the analysis.

**Table 3:** Multivariate risk factors associated with OAG.

Parameter	Odds Ratio (95% Confidence Interval)						*P V*alue
Men							
Age (year)	1	(	0.94	-	1.06	)	0.95
Self-report of hypertension	2.21	(	0.8	-	6.13	)	0.13
Have occupation	0.43	(	0.11	-	1.62	)	0.21
Exercise (hours/week)	1.07	(	0.98	-	1.15	)	0.11
Meat (eating days/week)	1.22	(	0.91	-	1.64	)	0.19
Intraocular pressure (mmHg/Ag)	1.2	(	1.05	-	1.38	)	0.009
Women							
Age (year)	0.99	(	0.93	-	1.04	)	0.72
Self-report of hypertension	1.74	(	0.66	-	4.61	)	0.26
Have occupation	0.77	(	0.27	-	2.14	)	0.61
Exercise (hours/week)	1.05	(	0.98	-	1.12	)	0.13
Meat (eating days/week)	0.61	(	0.43	-	0.88	)	0.007
Intraocular pressure (mmHg/Ag)	0.99	(	0.85	-	1.17	)	0.94
smoking (pack-year)	0.92	(	0.81	-	1.03	)	0.13

## Discussion

We conducted a population-based study in the rural northern Japanese city of Rumoi to identify what modifiable lifestyle risk factors are associated with the development of OAG. Our results indicated that a lower dietary intake of meat in women and high IOP in men were independent risks for OAG. In this study, our classification of meat included pork, beef, chicken and meat from other birds or animals. Fish consumption was assessed separately in the questionnaire. As pork is the most commonly consumed meat in Hokkaido prefecture (1.5 times more than chicken and 5 times more than beef: Family Income and Expenditure Survey by Ministry of Internal Affairs and Communications Statistics Bureau; http://www.stat.go.jp/data/kakei/5.htm), we speculate that consumption of pork, along with other meats, may reduce the risk of OAG in Japanese women. We assessed meat intake as number of days that meat was consumed per week and not as the amount of meat. So not the amount of eating meat but the frequency of eating meat may be important to reduce risk of OAG.

While we have found no studies investigating whether there is an association between OAG and diet in Asian countries, there are some reports of dietary risk factors from western counties. These include low consumption of nitrate, vegetables, peaches [12, 13], fatty fish, walnuts [7], retinol equivalents and vitamin B1 [17], with one study also identifying a higher omega 3:6 ratio intake as a risk factor for the development of OAG [18]. Whilst in the current study dietary meat intake was associated with OAG, there was no association with fruit and fish consumption. Because of the omega 3:6 ratio of pork, Japanese beef and chicken are lower than 0.1, and retinol equivalents and vitamin B1 are rich in pork (Standard tables of food composition in Japan 2015 by Ministry of Education, Culture, Sports, Science and Technology- Japan; http://www.mext.go.jp/en/policy/science_technology/policy/title01/detail01/sdetail01/1388555.htm), the results of the current study and the previous studies [17, 18] are not mutually exclusive.

In white populations, 30–39% of OAG cases are classified as normal tension glaucoma (NTG) [19–21]. However, the prevalence of NTG in Asian populations is significantly higher, with up to 85% of Japanese women with OAG classified as having NTG [22]. A lower dietary intake of meat may make the optic disc more susceptible to IOP-induced damage, which could contribute to the higher incidence of NTG in Asian populations, who traditionally eat less meat than those in western countries. In a Japanese population, Taniguchi Y et al. found an odds ratio of 2.06 (95% CI: 1.14–3.77; p < 0.05) for cognitive decline in the lowest tertiles of serum albumin (less than 4.1 g/dL) and they suggested that low nutritional status might have a negative impact on cognitive function [23]. Meat is one of best sources of serum albumin, a marker used to assess systemic nutritional status. A diet higher in meat consumption may also contribute to maintaining good nutritional status of the central nervous system [24].

The most established risk factors for OAG include high IOP, older age, myopia and a family history [7, 25]. In the current study, high IOP is a risk factor for OAG in men but not women. In the Tajimi study, the average IOP in individuals with OAG was 15.4 ± 2.8 mmHg in the right eye and 15.2 ± 2.8 mmHg in the left eye [26]. In participants without OAG, the average IOP was 14.5 ± 2.5 mmHg in the right eye and 14.4 ± 2.6 mmHg in the left eye[26]. In the current study, we observed a similar IOP measurement in the male cohort as what was found in the Tajimi study. However, as with most studies, the Tajimi study only reported on the IOP of a combined male and female cohort, so we could not assess if our finding of no association between IOP and OAG in the female population was unique.

Although we could not detect a statistical difference in age between the OAG and non-OAG participants, the mean age of the OAG participants was higher in both genders. We also stratified our analysis by gender to determine if specific risk factors were only associated with OAG in the male or female cohort. It has been reported that some risk factors for vascular and neuronal diseases are dependent on gender [27, 28] and there are only a few studies that have investigated stratified by gender with OAG [12, 29]. Therefore, more research is necessary to define what role gender has on OAG risk factors.

Whilst the combined male and female OAG group had significantly more systemic hypertension than the non-OAG group by univariate analysis, there was no significant difference by multivariate analysis (OR = 1.9; *p =* 0.07). A similar result was observed in the Tajimi study and the association between hypertension and OAG was speculated to be a caused by a correlation between age and hypertension[25]. As significantly more women in this study underwent a detailed examination, we could not draw conclusions on the role of gender with potential OAG risk factors.

In the current study, the smoking rate amongst the participants (39.8% in men, 12.8% in women) was higher than the smoking rate of Japanese adults (31.1% in men and 9.5% in women) reported in the Comprehensive Survey of Living Conditions 2016 (http://www.mhlw.go.jp/toukei/saikin/hw/k-tyosa/k-tyosa16/dl/04.pdf). This was particularly evident in older men (S1 Fig). While an association between heavy smoking (40 packs/year or more) and OAG has been reported [11], some studies have contradicted this finding [14, 30]. We observed a negative association between smoking and OAG in women by univariate analysis but not by multivariate analysis. No association was detected between OAG and smoking in men.

One third of the participants (45% of the men and 17% of the women) in this study who required a detailed examination did not undergo the examination, which could influence our findings. To account for this potential source of bias, we statistically compared the participants who underwent the detailed examination and those that did not. Significantly more men than women declined the opportunity to undergo a detailed examination (*p* < 0.001) and individuals who were employed were also less likely to attend the examination (*p* = 0.02; S5 Table). However, individuals who had higher rates of exercise were more likely to attend their examination appointment. In order to account for the variation in responses between men and women, we stratified our analysis of the OAG risk factors based on gender and adjusted the risk factors in the multivariate analysis. We also presented the findings from the univariate analysis to clarify the differences in the OAG analyses. We estimated that the prevalence of OAG in the participants who missed the detailed examination was similar to those who underwent the examination. Therefore, we concluded that the prevalence of OAG in the current study was 3.8%, which is similar to other Japanese prevalence data [26].

This study has some limitations. We recruited 11% of the residents from Rumoi city who were aged 40 years or older but they were not randomly selected. The participants needed to visit the Rumoi health station to take part in the screening examination. This excluded individuals who could not attend the clinic as a result of poor health. Additionally, no participants in this study were aged 90 years or older. These factors may have reduced the opportunity to identify an association between OAG and dementia. Another limitation of our study was that we did not assess all previously reported OAG risk factors, such as myopia and family history. Although family history can influence eating habits, meat consumption was the only dietary habit that we identified as having a relationship with OAG.

In conclusion, our results indicate that a higher weekly consumption of meat is negatively associated with OAG in Japanese women (OR = 0.61; 95% CI: 0.43–0.88; p = 0.007). A well balanced diet that includes meat consumption may contribute to reducing the risk of developing OAG.

## Supporting information

S1 Table Age distribution of the entire population of Rumoi city and the study participants. (PDF)

S2 Table Results of Fisher’s exact test comparing participants who underwent the detailed examination and those that did not. (PDF)

S3 Table Results of the Wilcoxon rank sum test comparing participants who underwent the detailed examination and those that did not. (PDF)

S4 Table Multivariate probability factors for undertaking a further examination. (PDF)

S5 Table Results of Fisher’s exact test comparing the OAG and non-OAG groups with smoking. (PDF)

S6 Table Appendix results of the Wilcoxon rank sum test comparing the OAG and non-OAG groups with estimated ocular perfusion pressure. (PDF)

S1 Text Questionnaire about life style. (PDF)

S1 Fig Smoking rate among the study participants and wider Japanese population. (PDF)
