# Supplementary material for: A low meat diet increases the risk of open-angle glaucoma in women—The results of population-based, cross-sectional study in Japan
Source: PLoS One. 2018 Oct 2;13(10):e0204955. doi: 10.1371/journal.pone.0204955 (PMC6168154; doi:10.1371/journal.pone.0204955)
Supplement: S1 Table — (PDF) [file pone.0204955.s001.pdf]

S1 Table. Age distribution of the entire population of Rumoi city and the study participants

Age Group (years)	participants			Entiire Population			Percetnage of Entire Population		
	Men	Women	All	Men	Women	All	Men	Women	All
40-49	312	153	465	1556	1424	2980	20.1	10.7	15.6
50-59	313	175	488	1537	1468	3005	20.4	11.9	16.2
60-69	247	244	491	1816	2184	4000	13.6	11.2	12.3
70-79	97	145	242	1389	1898	3287	7.0	7.6	7.4
80-89	20	25	45	668	1051	1719	3.0	2.4	2.6
90 and older	0	0	0	83	299	382	0	0	0
