# Supplementary material for: A low meat diet increases the risk of open-angle glaucoma in women—The results of population-based, cross-sectional study in Japan
Source: PLoS One. 2018 Oct 2;13(10):e0204955. doi: 10.1371/journal.pone.0204955 (PMC6168154; doi:10.1371/journal.pone.0204955)
Supplement: S2 Table — (PDF) [file pone.0204955.s002.pdf]

S2 Table. Results of Fisher's exact test comparing participants who underwent the detailed examination and those that did not

Parameter	Miss detailing examination n (%)	Took detailing examination n (%)	*P* Value
Total men and women	104 (100)	202 (100)	
Gender (male-femail)	84-20 (81-19)	102-100 (50-50)	0.0000002 **
Self-report of diabetes mellitus	10 (10)	25 (12)	0.57
Self-report of hypertension	26 (25)	74 (35)	0.04
Have occupation	85 (82)	107 (51)	0.0000005 **
Men	84 (100)	102 (100)	
Self-report of diabetes mellitus	8 (10)	14 (14)	0.50
Self-report of hypertension	20 (24)	30 (29)	0.41
Have occupation	71 (85)	73 (72)	0.05
Women	20 (100)	100 (100)	
Self-report of diabetes mellitus	2 (10)	11 (11)	1.00
Self-report of hypertension	6 (30)	44 (44)	0.32
Have occupation	14 (70)	34 (34)	0.005 **
