# Supplementary material for: A low meat diet increases the risk of open-angle glaucoma in women—The results of population-based, cross-sectional study in Japan
Source: PLoS One. 2018 Oct 2;13(10):e0204955. doi: 10.1371/journal.pone.0204955 (PMC6168154; doi:10.1371/journal.pone.0204955)
Supplement: S3 Table — (PDF) [file pone.0204955.s003.pdf]

S3 Table. Results of the Wilcoxon rank sum test comparing participants who underwent the detailed examination and those that did not

Parameter	Men			Women		
	Took detailing examination (n =102)	miss (n =84)	*P Value*	Took detailing examination (n =100)	miss (n =20)	*P Value*
Age (yrs)	61.1 10.7	57.5 ± 10.4	0.03 *	65.9 ± 9.9	62.8 ± 13.9	0.19
Questionnaire						
Number of family living together (include oneself)	2.5 ± 1.1	2.6 ± 1.1	0.24	2.2 ± 1.0	2.5 ± 1.2	0.27
Activity						
Walking（hours/day）	2.1 ± 2.1	1.6 ± 1.5	0.29	2.4 ± 1.9	4.3 ± 4.3	0.13
Exercise（hours/week）	2.8 ± 5.1	1.4 ± 2.5	0.04 *	2.5 ± 3.7	1.5 ± 2.7	0.14
Habits						
Smoking（pack-year）	30 ± 33	23 ± 23	0.26	4 ± 9	3 ± 8	0.99
Coffee（cups/day）	1.9 ± 1.8	2.3 ± 2.5	0.50	1.6 ± 1.7	2.1 ± 1.3	0.11
Tea (cups/day)	1.6 ± 1.8	1.3 ± 1.7	0.12	2.5 ± 2.3	1.5 ± 1.6	0.08
Alcohol （glasses/day）	1.4 ± 2.7	1.2 ± 1.4	0.46	0.3 ± 0.7	0.3 ± 0.7	0.69
Fruit (number/day)	0.7 ± 0.7	0.6 ± 0.6	0.19	1.0 ± 0.8	1.1 ± 0.8	0.69
Meat (eating days/week)	2.3 ± 1.4	2.5 ± 1.5	0.54	2.1 ± 1.4	2.6 ± 1.2	0.11
Fish (eating days/week)	3.4 ± 1.8	3.0 ± 1.6	0.15	3.9 ± 1.8	3.6 ± 1.4	0.36
Measurments						
Body mass index (kg/m^2)	27.4 ± 25.4	25.5 ± 3.8	0.32	23.8 ± 3.9	24.5 ± 4.4	0.51
Systolic blood pressure(mmHg/Ag)	136 ± 18	135 ± 23	0.40	132 ± 18	134 ± 16	0.55
Diastolic pressure(mmHg/Ag)	79.9 ± 11.3	78.9 ± 12.6	0.44	76.5 ± 11.5	81.0 ± 14.3	0.29
Pulse rate/minute	75.4 ± 11.8	78.1 ± 14.3	0.21	74.9 ± 10.9	78.3 ± 13.6	0.40
Intraocular pressure (mmHg/Ag)						
left eye	14.9 ± 3.4	14.9 2.9	0.77	14.1 2.9	15.0 2.6	0.15
right eye	15.0 ± 3.0	14.7 2.8	0.49	14.2 2.9	15.0 2.9	0.20
	(mean ± SD)	(mean ± SD)		(mean ± SD)	(mean ± SD)	
