# Supplementary material for: A low meat diet increases the risk of open-angle glaucoma in women—The results of population-based, cross-sectional study in Japan
Source: PLoS One. 2018 Oct 2;13(10):e0204955. doi: 10.1371/journal.pone.0204955 (PMC6168154; doi:10.1371/journal.pone.0204955)
Supplement: S4 Table — (PDF) [file pone.0204955.s004.pdf]

S4 Table. Multivariate probability factors for undertaking a further examination

Parameter	Odds Ratio	(95% Confidence Interval)	*P Value*
**Total men and Women**			
Gender (women)	2.91	(1.59 - 5.32)	0.0005**
Age (year)	1.00	(0.97 - 1.03)	0.99
Self-report of hypertension	1.33	(0.73 - 2.41)	0.35
Have occupation	0.43	(0.21 - 0.87)	0.02*
Exercise (hours/week)	1.08	(0.99 - 1.19)	0.08
Meat (eating days/week)	0.88	(0.73 - 1.06)	0.19
Intraocular pressure (mmHg/Ag)	0.97	(0.89 - 1.06)	0.49
**Men**			
Age (year)	1.01	(0.98 - 1.05)	0.46
Self-report of hypertension	1.21	(0.60 - 2.44)	0.59
Have occupation	0.69	(0.29 - 1.69)	0.42
Exercise (hours/week)	1.09	(0.98 - 1.21)	0.10
Meat (eating days/week)	0.93	(0.75 - 1.16)	0.53
Intraocular pressure (mmHg/Ag)	1.00	(0.91 - 1.10)	0.99
**Women**			
Age (year)	0.97	(0.92 - 1.03)	0.32
Self-report of hypertension	1.64	(0.51 - 5.24)	0.41
Have occupation	0.17	(0.05 - 0.62)	0.007**
Exercise (hours/week)	1.07	(0.90 - 1.28)	0.45
Meat (eating days/week)	0.75	(0.51 - 1.10)	0.14
Intraocular pressure (mmHg/Ag)	0.88	(0.73 - 1.05)	0.15
