# Supplementary material for: A low meat diet increases the risk of open-angle glaucoma in women—The results of population-based, cross-sectional study in Japan
Source: PLoS One. 2018 Oct 2;13(10):e0204955. doi: 10.1371/journal.pone.0204955 (PMC6168154; doi:10.1371/journal.pone.0204955)
Supplement: S5 Table — (PDF) [file pone.0204955.s005.pdf]

S5 Table. Results of Fisher's exact test comparing the OAG and non-OAG groups with smoking

Parameter	OAG n (%)	non-OAG n (%)	*P* Value
Male	19 (100)	861 (100)	
smoking history; present-past-non	8-4-7 (42-21-37)	347-336-178 (40-39-21)	0.13
smoking 40 pack-year or more	2 (11)	154 (18)	0.55
Female	23 (100)	680 (100)	
smoking history; present-past-non	1-2-20 (4-9-87)	90-102-488 (13-15-72)	0.38
smoking, 40 pack-year or more	0 (0)	14 (2)	1.00
