# Supplementary material for: A low meat diet increases the risk of open-angle glaucoma in women—The results of population-based, cross-sectional study in Japan
Source: PLoS One. 2018 Oct 2;13(10):e0204955. doi: 10.1371/journal.pone.0204955 (PMC6168154; doi:10.1371/journal.pone.0204955)
Supplement: S6 Table — (PDF) [file pone.0204955.s006.pdf]

S6 Table. Results of the Wilcoxon rank sum test comparing the OAG and non-OAG groups with estimated ocular perfusion pressure

	minimum	25%	median	75%	maximum	*P* value
men						
OAG	42.4	45.0	52.3	56.0	65.5	0.97
non-OAG	28.6	45.7	50.6	56.4	88.7	
women						
OAG	32.6	43.6	48.1	50.3	65.5	0.67
non-OAG	24.0	42.9	47.8	53.8	79.6	

Estimated ocular perfusion pressure= 2/3 [DBP + 1/3(SBP – DBP)] – IOP
DBP: Diastolic blood pressure SBP: Systolic blood pressure
IOP: intraocular pressure
Reference ; Mitra, S. et al. Invest Ophthalmol Vis Sci 2005;46(2):561–567
