# Supplementary material for: A low meat diet increases the risk of open-angle glaucoma in women—The results of population-based, cross-sectional study in Japan
Source: PLoS One. 2018 Oct 2;13(10):e0204955. doi: 10.1371/journal.pone.0204955 (PMC6168154; doi:10.1371/journal.pone.0204955)
Supplement: S1 Text — (PDF) [file pone.0204955.s007.pdf]

S1 Text − Questionnaire about life style -

Name ________ Age ______ sex

Occupation _______________

Do you have smoking habit? __________

How many cigarettes do you smoke?

________ (number) for ________ years

Did you used to smoke?

_________ (number) for ________ years

How many hours do you walk in everyday life? (include walking as exercise)

______ hours

What kind of exercise do you do, and how many hours do you do it a week?

I do ________ for _____ hours a week for exercise.

How many people are you living together, including yourself? ________

<Eating and dringking habit>

Coffee ___ cups a day

Green tee __ cups a day

Alcohol ___ cups of _____ a day

Fruit ___ (number) a day

How many days do you eat meat a week?

___ days a week

How many days do you eat fish a week?

___ days a week
