# Supplementary figures and images for: A low meat diet increases the risk of open-angle glaucoma in women—The results of population-based, cross-sectional study in Japan
Source: PLoS One. 2018 Oct 2;13(10):e0204955. doi: 10.1371/journal.pone.0204955 (PMC6168154; doi:10.1371/journal.pone.0204955)

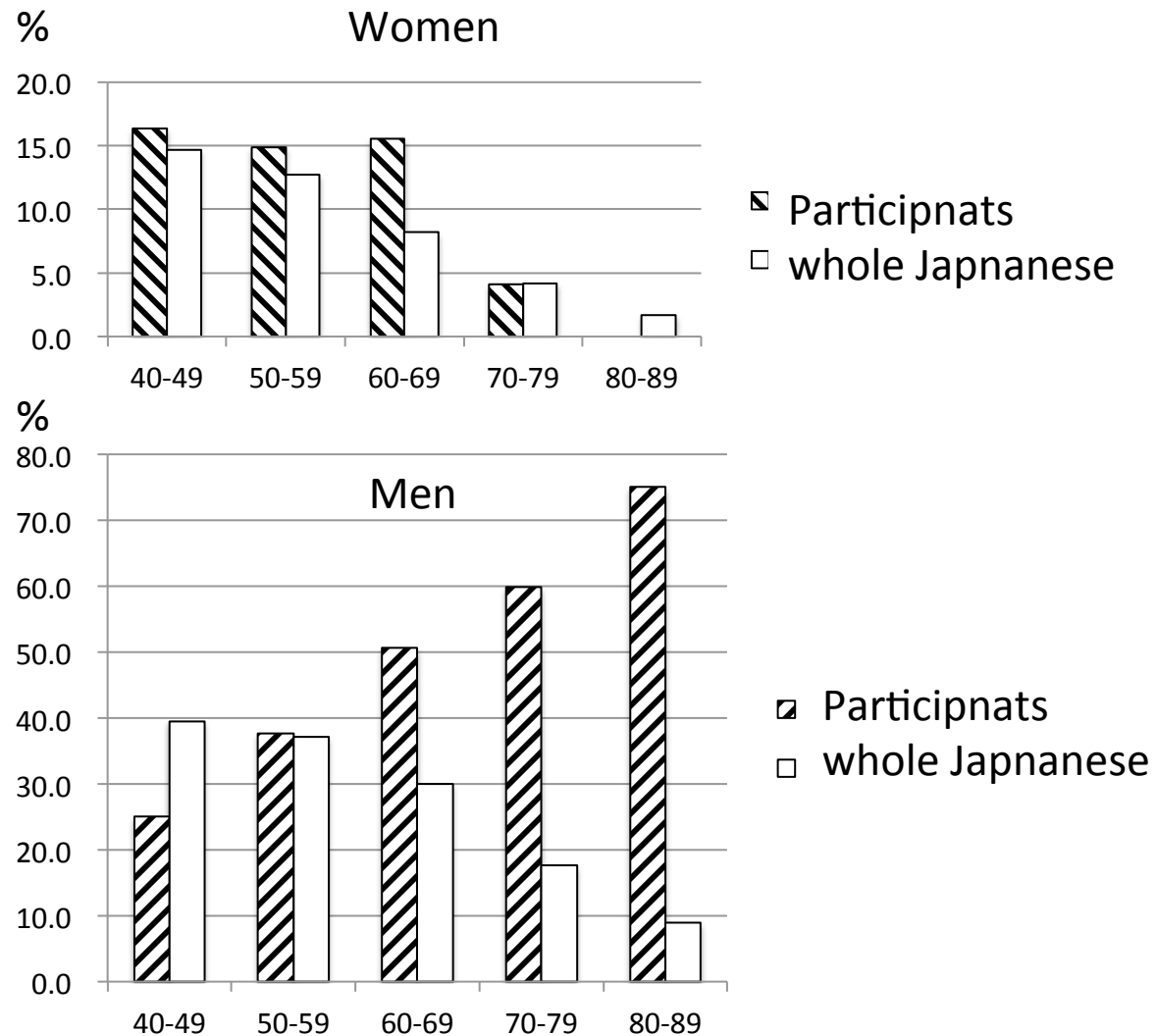

S1 Fig. Smoking rate among the study participants and wider Japanese population

Supplement: S1 Fig — (PDF) [file pone.0204955.s008.pdf]
